# Current management of fluid balance in critically ill patients with acute kidney injury: A scoping review

**DOI:** 10.1016/j.ccrj.2023.06.002

**Published:** 2023-07-27

**Authors:** Kyle C. White, Ahmad Nasser, Michelle L. Gatton, Kevin B. Laupland

**Affiliations:** a Intensive Care Unit, Princess Alexandra Hospital, Woolloongabba, Queensland, Australia; b Faculty of Health, Queensland University of Technology (QUT), Brisbane, Queensland, Australia; c Intensive Care Unit, Queen Elizabeth II Jubilee Hospital, Coopers Plains, Queensland, Australia; d Faculty of Medicine, University of Queensland, Herston, Queensland, Australia; e Department of Intensive Care Services, Royal Brisbane and Women's Hospital, Brisbane, Queensland, Australia

**Keywords:** Anaesthesia and intensive care, Intensive care, Renal replacement therapy, Acute kidney injury, Fluid management

## Abstract

**Objective:**

The overall objective of this scoping review is to assess the extent of the literature related to the fluid management of critically ill patients with acute kidney injury (AKI).

**Introduction:**

AKI is common in critically ill patients where fluid therapy is a mainstay of treatment. An association between fluid balance (FB) and adverse patient-centred outcomes in critically ill patients with AKI regardless of severity has been demonstrated. The evidence for the prospective intervention of FB and its impact on outcomes is unknown.

**Inclusion criteria:**

All studies investigating FB in patients with AKI admitted to an intensive care unit were included. Literature not related to FB in the critically ill patient with AKI population was excluded.

**Methods:**

We searched MEDLINE, EMBASE, and CINAHL from January 1st, 2012, onwards. We included primary research studies, experimental and observational, recruiting adult participants admitted to an intensive care unit who had an AKI. We extracted data on study and patient characteristics, as well as FB, renal-based outcomes, and patient-centred outcomes. Two reviewers independently screened citations for eligible studies and performed data extraction.

**Results:**

Of the 13,767 studies reviewed, 22 met the inclusion criteria. Two studies examined manipulation of fluid input, 18 studies assessed enhancing fluid removal, and two studies applied a restrictive fluid protocol. Sixteen studies examined patients receiving renal replacement therapy, five studies included non–renal replacement therapy patients, and one study included both. Current evidence is broad with varied approaches to managing fluid input and fluid removal. The studies did not demonstrate a consensus approach for any aspect of the fluid management of critically ill patients. There was a limited application of a restrictive fluid protocol with no conclusions possible.

**Conclusions:**

The current body of evidence for the management of FB in critically ill patients with AKI is limited in nature. The current quality of evidence is unable to guide current clinical practice. The key outcome of this review is to highlight areas for future research.

## 1 Introduction

Acute kidney injury (AKI) is common in the critically ill population, occurring in 35–50% of patients admitted to the intensive care unit (ICU).^1^^,^^2^ Individuals who develop an AKI in the ICU have hospital mortality of more than 30%, the magnitude of which increases with the severity of AKI.^1^^,^^3^

Conventional management for AKI is the administration of fluids to improve renal perfusion;^4^ however, there is uncertainty surrounding the universality of this practice.^5^^,^^6^ A prospective analysis of oliguric ICU patients who received a physician-directed 500 -mL fluid bolus demonstrated only 50% of patients had an increase in urine output (UO).^7^ Patients with severe sepsis who received a fluid bolus triggered by reduced UO did not have an alteration in their UO.^8^

There is a growing body of observational evidence that fluid overload is associated with harm among patients with AKI. In critically ill patients with AKI not requiring renal replacement therapy (RRT), a positive FB at diagnosis of AKI and at 48 h was independently associated with renal non-recovery.^3^ Furthermore, a higher cumulative fluid balance (FB) at 72 h was independently associated with an increased risk of 28-day mortality in a large multicentre prospective cohort.^9^

In critically ill patients undergoing RRT, the presence of fluid overload as defined by percentage weight gain at the time of RRT initiation has been associated with an increased risk of RRT dependence at 1 year.^10^ In addition, a post hoc analysis of the Randomized Evaluation of Normal versus Augmented Level (RENAL) study^11^ cohort demonstrated that a positive cumulative FB after RRT initiation was associated with increased mortality.^12^ Strengthening the association, a single-centre retrospective study of 399 patients who underwent RRT demonstrated that a net negative FB during the first 72 h of RRT was independently associated with lower hospital mortality.^13^

As demonstrated above, there is a recurrent association between fluid management and adverse patient-centred outcomes in critically ill patients with AKI regardless of severity. However, given the observational nature of the evidence, the cause-and-effect relationship between FB and outcomes remains undefined. It is unknown if manipulating FB in critically ill patients with AKI affects patient outcomes, and furthermore, there is uncertainty as to which methods of manipulating FB significantly alter the fluid state. This scoping review aims to systematically assess the extent of the current literature related to the management of FB in critically ill patients with AKI. Our objective is to determine current evidence-based interventions or identify areas for future research.

## 2 Review questions

What is the current body of evidence for management of FB in critically ill patients with AKI?

1. What is the current evidence for the manipulation of fluid input?
2. What is the current evidence for enhancing fluid removal?
3. What is the current evidence for comprehensive fluid protocols that have a multimodal approach towards FB management?

## 3 Inclusion criteria

### 3.1 Participants

All studies investigating an FB intervention in adult patients with AKI admitted to an ICU were included. Literature not directly related to FB in our target population was excluded.

### 3.2 Types of sources

This scoping review considered both experimental and quasi-experimental study designs. In addition, analytical observational studies including prospective and retrospective cohort studies were considered for inclusion. All of the studies were not considered for inclusion in this scoping review.

## 4 Methods

The scoping review was conducted in accordance with the Joanna Briggs Institute methodology for scoping reviews.^14^

### 4.1 Search strategy

The search strategy, as demonstrated in the **Online Supplement**, aimed to locate both published and unpublished studies. A systematic search was performed in MEDLINE, CINAHL, and EMBASE databases. The core concepts of the search strategy included critical illness, AKI, and FB. Studies published since January 1st, 2012, were included as intensive care fluid management has changed gradually over the past decade.^15^ Further, the date of 2012 coincides with the introduction of the Kidney Disease Improving Global Outcomes (KDIGO) definition of AKI.^16^ The reference list of included articles, as well as a grey literature search including general and targeted website searching, was performed.

### 4.2 Study/source of evidence selection

All identified citations were uploaded into Covidence for review (Covidence systematic review software, Veritas Health Innovation, Melbourne, Australia; available at www.covidence.org). Initially, titles and abstracts and, subsequently, full text of selected citations were assessed against the inclusion criteria by two independent reviewers (A.N. and K.W.). The reasons for exclusion of full-text reviewed articles were recorded for reporting. Any disagreements that occurred at each stage of the selection process were resolved through discussion.

### 4.3 Data extraction

Data were extracted from included articles by two independent reviewers. The data extracted included specific details about the participants, study methods, details of intervention, FB details, and additional key findings relevant to the review questions.

## 5 Results

### 5.1 Search results

**Fig. 1:**
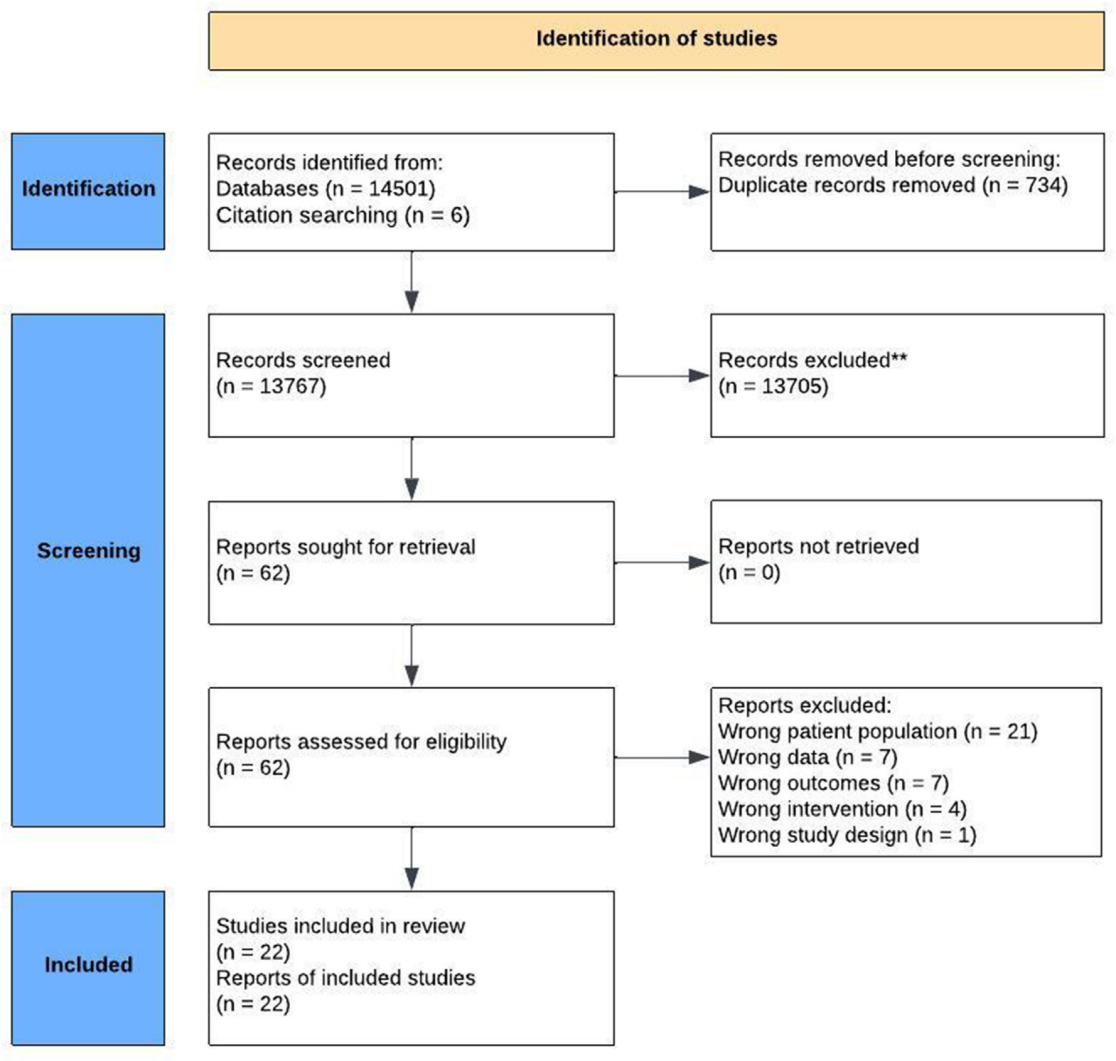
PRISMA flow diagram.

The search strategy resulted in 14,507 articles. After the removal of 734 duplicates, the title and abstract of 13,767 studies were screened. After screening, 62 full-text articles were assessed, resulting in 22 included studies. The Preferred Reporting Items for Systematic Reviews and Meta-Analyses (PRISMA) flow diagram is shown in Fig. 1.

### 5.2 Included studies and participants

Overall, 22 studies involving 23,970 patients were included in the scoping review.[17], [18], [19], [20], [21], [22], [23], [24], [25], [26], [27], [28], [29], [30], [31], [32], [33], [34], [35], [36], [37], [38] Two studies investigated fluid input,^17^^,^^19^ 17 studies investigated fluid removal,^18^^,^[20], [21], [22], [23], [24], [25], [26], [27], [28], [29], [30], [31], [32], [33]^,^^36^^,^^37^ and two studies examined a restrictive fluid protocol (RFP).^34^^,^^35^ Five studies were conducted in a population not receiving RRT,^17^^,^[20], [21], [22]^,^^35^ 16 studies investigated AKI patients on RRT,^18^^,^^19^^,^[23], [24], [25], [26], [27], [28], [29], [30], [31], [32]^,^^34^^,^[36], [37], [38] and one study included both.^33^ Of note, one retrospective study was responsible for a majority of the cohort with 14,151 patients.^22^ Of the included studies, eight were observational,^21^^,^^22^^,^[26], [27], [28]^,^^32^^,^^34^^,^^36^ nine were interventional,^17^^,^^18^^,^^20^^,^^23^^,^^25^^,^^29^^,^^33^^,^^35^^,^^38^ and five were post hoc analyses of interventional trials.^19^^,^^24^^,^^30^^,^^31^^,^^37^ Ten of the studies were single centre,^17^^,^^18^^,^^21^^,^^23^^,^^26^^,^^28^^,^^29^^,^^32^^,^^34^^,^^36^ and 12 were multicentre.^19^^,^^20^^,^^22^^,^^24^^,^^25^^,^^27^^,^^30^^,^^31^^,^^33^^,^^35^^,^^37^^,^^38^

### 5.3 Manipulation of fluid input

As shown in Table 1, two studies with a total of 811 patients investigated the manipulation of fluid input.^17^^,^^19^ In non-RRT patients, Effat et al. demonstrated less fluid input after 48 h with echocardiography-derived stroke volume variation–guided fluid compared to central venous pressure–guided fluid in 40 patients. In RRT patients, O'Brien et al. retrospectively examined the use of different types and combinations of albumin solutions (20% albumin vs 4% albumin vs both) in the RENAL trial,^11^ demonstrating in an unadjusted analysis a lower daily FB and cumulative FB with 20% albumin than with 4% albumin and both. No studies have solely investigated the impact of a restrictive fluid input strategy based on FB or patient-centred outcomes.

**Table 1:** Manipulation of fluid input included studies.

Reference	Study Design	Country	Sample Size	AKI Definition	Population	RRT	Intervention	Comparator	Assessment of Fluid Status	Duration of Intervention	Difference in FB	Outcome Measures
Effat 2021	SC RCT, Unblinded	Egypt	40 (20/20)	Unclear	AKI, ICU	Non-RRT patients	Echo-directed SVV fluid administration	CVP-directed fluid	Incomplete	48 h	Fluid input (2200 mL vs 3700 mL; p < 0.01). No UO or FB data.	ICU LOS (6.4 days vs 12.1 days; p = 0.183). Mortality (10% vs 40%; p = 0.028).
O'Brien 2022	Post hoc analysis of RENAL MC RCT	Australia & New Zealand	771	On CRRT.	ICU; CRRT	RRT as inclusion criteria	1) 20% albumin 2) 20% and 4% albumin	4% albumin	Cumulative fluid balance	Until CRRT cessation or ICU discharge	Daily FB (20% HAS 288 mL, 4% HAS 245 mL, combined HAS 88 mL (p < 0.001). Cumulative FB (1.9 L vs −2.4 vs 1.3; p < 0.01).	Mortality at day 90 (4% HAS 48% vs 20% HAS 52% vs Combined HAS 51%; p = 0.65); RRT dependence at day 90 (6.7% vs 5.3% vs 3.8%; p = 0.59).

Abbreviations: AKI = acute kidney injury; SC = single-centre; MC = multicentre; RCT = randomised controlled trial; CRRT = continuous renal replacement therapy; RRT = renal replacement therapy; ICU = intensive care unit; FB = fluid balance; UO = urine output; RENAL = Randomized Evaluation of Normal versus Augmented Level; HAS = human albumin solution; LOS = length of stay; CVP = central venous pressure.

### 5.4 Manipulation of fluid output

Fluid removal was investigated in 18 studies involving 22,952 critically ill patients with AKI.^18^^,^[20], [21], [22], [23], [24], [25], [26], [27], [28], [29], [30], [31], [32], [33]^,^^36^^,^^38^ Fifteen studies were conducted in patients receiving RRT,^18^^,^[23], [24], [25], [26], [27], [28], [29], [30], [31], [32], [33]^,^[36], [37], [38] whereas only three studies involved patients not receiving RRT.[20], [21], [22] An overview of the included studies examining fluid output is shown in Table 2.

**Table 2:** Manipulation of fluid output included studies.

Reference	Study Design	Country	Sample Size	AKI Definition	Population	RRT	Intervention	Comparator	Assessment of Fluid Status	Duration of Intervention	Difference in FB	Outcome Measures
Non-RRT patients												
Bagshaw 2017	MC Pilot RCT	Canada & Australia	73 (37/36)	RIFLE	AKI, RIFLE - RISK; ICU; CVL; IDC; SIRS = 2+; ‘Resuscitated'	Non-RRT patients	Furosemide bolus & infusion; titrated to UO	Placebo	Fluid balance	7 days; until RRT, death, or discharge	877 mL vs 2407 mL (- 1081 mL; 95% CI, −2697 to 467)	Worsening AKI (43.2% vs. 37.1%, p = 0.6); Kidney recovery (29.7% vs. 42.9%, p = 0.3); RRT (27.0% s. 28.6%, p = 0.8)
Cagliani 2021	SC Retrospective	USA	126 (39/87)	RIFLE	AKI, RIFLE - R, I or F; ICU; Surgical	Non-RRT patients	Furosemide & fenoldopam	Furosemide alone	Fluid balance	24 h	R group: 1588 mL [774 mL–3765mL] vs 1074 mL [612 mL–3996mL]; p = 0.07. I or F group: 5179 mL [2121 mL–7233mL] vs 4230 mL [2132 mL–7843 mL]; p = 0.06.	UO (421 mL vs 320 mL; p = 0.22); FB (4230 mL vs 5179 mL; p = 0.06); CrCl (48.5 vs 40.7; p = 0.53).
Zhao 2020	MC Retrospective	USA	14154 (7885/6269)	KDIGO	ICU; AKI	Non-RRT patients	Furosemide	No diuretic	Fluid balance	Variable	Net FB (−575 mL vs −562 mL; p = 0.5); Positive FB (33.4% vs 33%; p = 0.752).	Increase AKI stage (HR 1.13; 95% CI 1.05–1.21; p < 0.01); RRT (HR 1.5; 95% CI 1.29–1.76; p < 0.01); In-hospital mortality (12.7% vs 21.7%; p < 0.01).
**RRT Patients**												
Nuchpramool 2019	SC RCT	Thailand	36 (17/19)	On CRRT.	ICU; CRRT	RRT as inclusion criteria	BIVA-guided fluid management	Standard care	BIVA & fluid accumulation (unclear if FB or weight-based)	Not provided	Details not provided. “ … did not provide beneficial effects in the rate of fluid removal indicating by body weight and %FA."	Mortality at day 28 (47% vs 52.6%; p = 0.78)
RashidFarokhi 2022	SC RCT	Iran	65 (32/33)	Not provided. On CRRT.	ICU; CRRT	RRT as inclusion criteria	BIVA-guided, UFnet prescription	Clinical parameters guided UFnet prescription	Bioimpedance vector analysis (BIVA)	∼50 h.	BIVA-defined hypervolaemia (31.3 vs 63.6%, p = 0.009)	LBM (80.7 vs. 85.9; OR: 5.2; 95% CI: 0.2 to 10.1; p < 0.05); UO (0.9 mL/kg/hr; OR: 0.6; 95% CI: 0.4–1.1; p = 0.04); Mortality at 30 days (53.15 vs 60.6%; OR: 0.7; 95% CI: 0.3–2; p = 0.54)
Shin 2021	SC Retrospective	South Korea	216 (88/42)	Not provided	ICU; CRRT	RRT as inclusion criteria	<20% downtime	≥ 20% downtime	Fluid balance	Not provided	Not provided. Daily fluid balance lower on day 2 (p = 0.046) and day 3 (p = 0.031).	Mortality (p = 0.95).
Mishra 2017	SC RCT	India	60 (30/30)	KDIGO	ICU; CRRT; AKI; Septic shock	RRT as inclusion criteria	SLED	CRRT	Fluid balance	Median 3.5 days	Fluid balance per 24 h (0.79 L vs 0.68; p = 0.10)	Haemodynamic stability, delta VD (39 vs 42; p = 0.39)
McCausland 2016	Post-hoc analysis of ATN MC RCT	USA	871 (436/435)	On CRRT.	ICU; AKI; CRRT	RRT as inclusion criteria	Less intense dialysis (IHD or SLED 3x/week or CRRT at 20 mL/kg/hr)	Intensive dialysis (IHD or SLED 6x/week or CRRT at 35 mL/kg/hr)	Fluid balance	5-6 treatments	Net balance (- 200 mL vs 4 mL; p < 0.01)	UO (159 mL vs 106 mL; p < 0.01); UF volume (1850 mL vs 1700 mL; p = 0.22)
Gaudry 2018	Post-hoc analysis of AKIKI MC RCT	France	348 (174/174)	On CRRT.	ICU; Septic shock; AKI;	RRT as intervention	Early RRT (post-randomisation)	Late RRT (specific criteria to commence)	Fluid balance	Until CRRT cessation or ICU discharge	FB first 48 h (2.2 L vs 2 L; p = 0.93); Fluid input first 48 h (4.1 L vs 4.1 L; p = 0.55); UO first 48 h (994 mL vs 1881 mL; P < 0.001); UF first 48 h (3.6 L vs 2.1 L; p < 0.01).	RRT dependence at 60 days (3% vs 3%; p = 0.62)
Wald 2015	Pilot MC RCT	Canada	101 (48/52)	Not provided. On CRRT.	ICU; CRRT	RRT as inclusion criteria	Accelerated RRT (less than 12 h)	Standard Care	Fluid balance	14 days	FB at day 14 (−1336 mL vs −57 mL)	Mortality at day 90 (38% vs 37%; p = 0.92)
Xing 2019	SC Retrospective	China	141 (57/84)	RIFLE	ICU; Septic AKI; CVP data	RRT as inclusion criteria	Early initiation (within 12 h of F criteria)	Delay initiation (delay 48 h from F criteria)	Fluid balance	5 days; death or discharge	FB (1402 mL vs 1543 mL; p = 0.65)	FB (as shown)
Wald 2022	Post-hoc analysis of START-AKI MC RCT	Multinational	2716 (1366/1350)	KDIGO	ICU; AKI; CRRT	RRT as inclusion criteria	Accelerated strategy for CRRT initiation	Standard strategy	Fluid balance	14 days	FB at 14 days (4509 mL vs 5646 mL; p = 0.03)	Mortality at 90 days by FB quartiles 1–4 (1 = 40% vs 2 = 45.5% vs 3 = 45.2% vs 4 = 44.9%; p = 0.17)
Gaudry 2021	MC RCT	France	278 (137/141)	KDIGO	ICU; AKI; CRRT	RRT as inclusion criteria	Delayed strategy	More delayed strategy	Fluid balance	Until CRRT cessation of ICU discharge	FB at 2 days (1584 mL vs 1581 mL; p 0.99); FB at 7 days (1744 mL vs 2072 mL; p = 0.79)	RRT-free days at 60 days (12 vs 10; p = 0.93)
Murugan 2018	SC Retrospective	USA	1075 (475/166/434)	On CRRT.	ICU; AKI; CRRT	RRT as inclusion criteria	Net ultrafiltration rate	Different rates (mL/kg/day): 1) <20, 2) 20 - <25 3) >25	Fluid balance adjusted to body weight	Median 4.7–8.7 days	Cumulative FB at day 7 (10.1 vs 10.5 vs 10.1; p = 0.78); UF (19.5 L vs 27.9 L vs 26.6 L; p < 0.01); FB excluding UF (13.5 L vs 22 L vs 19 L; p < 0.01).	Renal recovery at 1 year in survivors (82.6% vs 72.7% vs 78.4%; p = 0.25); Mortality at 1 year (69.7% vs 60.2% vs 59.4%; p = 0.003)
Murugan 2019	Post-hoc analysis of RENAL MC RCT	Australia & New Zealand	1434 (477/479/478)	On CRRT.	ICU; CRRT	RRT as inclusion criteria	Net ultrafiltration rate	Different rates (mL/kg/hr): 1) <1.01, 2) 1.01–1.75 3) >1.75	Fluid balance	Until CRRT cessation or ICU discharge	Cumulative FB (2.3 L vs −0.4 L vs −3.6 L; p < 0.01). NUF (1.7 L vs 8.5 L vs 16.5 L; p < 0.01); FB excluding NUF (4.6 L vs 8.5 L vs16.5 L; p < 0.01)	RRT dependence at day 90 (3.8% vs 5.8% vs 6.9%; p = 0.28); Mortality at day 90 (44.9% vs 39.2% vs 48.6%; p = 0.01)
Naorungroj 2020	SC Retrospective	Thailand	347 (159/102/86)	On CRRT	ICU; CRRT	RRT as inclusion	Net ultrafiltration	Different rates (mL/kg/hr): 1) <1.01, 2) 1.01–1.75 3) >1.75	Fluid balance	Until CRRT cessation or ICU discharge	Cumulative FB (527.0 mL vs −657 mL vs −1751 mL; p < 0.01);	Unadjusted RRT dependence at day 28 (21.3% vs 17.9% vs 18.4%; p = 0.66); Unadjusted mortality at day 28 (25.8% vs 32.7% vs 18.4%; p = 0.066).
Wu 2021	MC Retrospective	USA	911 (165/369/377)	Not provided. On CRRT.	ICU; Sepsis; CRRT	RRT as inclusion criteria	Net ultrafiltration rate	Different rates (mL/kg/hr): 1) <1.6, 2) 1.6–3.1, 3) >3.1	Fluid overload expressed as percentage	Until CRRT cessation or ICU discharge	Cumulative fluid overload in 48 h (−0.6% vs −2.8% vs −4.8%; p < 0.01)	In-hospital mortality (53.3% vs 40.4% vs 44.3%; p = 0.021); Renal recovery (55.2% vs 71.0% vs 69.5%; p < 0.01).
**Non-RRT and RRT Patients**												
Berthelsen 2018	MC Pilot RCT	Denmark	20 (7/13)	Renal Recovery Score (RRS)	RRS, Mod-High Risk (<60%); ICU <24 hrs; 10% fluid accumulation	RRT commenced as per protocol	Forced fluid removal to achieve CFB <1000 mL. Furosemide bolus & infusion then CRRT.	Standard care	Fluid balance	5 days; death or discharge	- 8434 mL vs - 641 mL (5814 mL; 95% CI: 2063–9565, P = 0.003)	CFB (as shown); Mean DFB (−1269 mL vs 133 mL; p < 0.01); Achieve neutral FB (86% vs 30%; p = 0.06)

Abbreviations: AKI = acute kidney injury; SC = single centre; MC = multicentre; RCT = randomised controlled trial; CRRT = continuous renal replacement therapy; RRT = renal replacement therapy; ICU = intensive care unit; FB = fluid balance; UO = urine output; CFB = cumulative fluid balance; CVL = central venous line; IDC = indwelling catheter; SIRS = systemic inflammatory response syndrome; CI = confidence interval; NUF = net ultrafiltration rate; STARRT-AKI = STandard versus Accelerated initiation of Renal Replacement Therapy in Acute Kidney Injury; ATN = Acute Renal Failure Trial Network; AKIKI = Artificial Kidney Initiation in Kidney Injury; RENAL = Randomized Evaluation of Normal versus Augmented Level; BIVA = bioimpedance vector analysis.

In the non-RRT population, three studies investigated the impact of medications on FB and outcomes. In a pilot randomised clinical trial (RCT), Bagshaw compared furosemide to placebo in resuscitated critically ill patients, demonstrating a lower FB with furosemide at 7 days.^20^ Zhao et al. retrospectively examined a furosemide versus no furosemide strategy and, at 5 days, did not demonstrate a difference in net FB or incidence of positive FB.^22^ Cagliani retrospectively compared fenoldopam with and without furosemide in 126 critically ill surgical patients and demonstrated the addition of fenoldopam did not result in a statistically significant difference in FB.^21^

In the RRT population, two studies directly examined the alteration of fluid removal via RRT, both of which utilised bioimpedance vector analysis (BIVA) guidance. Nuchpramool et al. demonstrated no difference in FB with BIVA-guided fluid management compared to standard care in 36 patients receiving RRT.^23^ In a single-centre RCT by Rashid-Farokhi et al., patients randomised to BIVA-guided ultrafiltration prescription compared to clinical parameter–guided ultrafiltration prescription had a lower rate of hypervolaemia, as well as a high ultrafiltration rate and higher UO.^18^

The indirect impact that different variables of RRT administration had on FB was investigated in 12 studies. Given the integral role of RRT in the management of FB, the manipulation of one aspect of the delivery of RRT would impact FB. Four studies that investigated the timing of initiation of RRT also reported on the impact on FB.[24], [25], [26]^,^^37^ Wald et al., in a pilot RCT^25^ and in a post hoc analysis of the STandard versus Accelerated initiation of Renal Replacement Therapy in Acute Kidney Injury trial,^37^ demonstrated a significant difference in FB at 14 days with early initiation. In contrast, Xing et al.,^26^ in a retrospective study, and Gaudry et al.,^24^ in a post hoc analysis of AKIKI RCT,^39^ did not find a difference in FB with early commencement of RRT at 5 days or 48 h, respectively. Gaudry et al. examined delayed and more delayed RRT initiation strategy and found no difference in FB at 7 days.^38^

Shin et al. retrospectively examined the impact of RRT downtime in critically ill patients undergoing RRT and demonstrated a lower daily FB on day 2 with less downtime.^28^ Mishra et al. compared sustained low-efficiency dialysis (SLED) to continuous renal replacement therapy in critically ill patients with septic shock and demonstrated no difference in FB.^29^ Mc Causland et al. performed a post hoc analysis of the acute renal failure trial network trial^40^ and demonstrated the less intensive dialysis group had a lower net FB.^30^

Four studies examined the impact of different net ultrafiltration (NUF) rates on patient-centred outcomes.^27^^,^^31^^,^^32^^,^^36^ Given that NUF is the removal of fluid via RRT, NUF rates are intertwined with FB. Murugan et al., in a post hoc analysis of the RENAL RCT,^11^^,^^32^ and Naorungroj et al., in a single-centre retrospective study,^36^ both demonstrated a lower cumulative FB with higher NUF rates. In contrast, the retrospective study performed by Murugan et al. showed no difference in cumulative FB with higher NUF rates.^31^ Wu et al. demonstrated that NUF rates impact resolution of fluid overload at 48 h of therapy.^27^ Of note, all four of the mentioned NUF studies did demonstrate worse patient-centred outcomes with the high NUF rate.

One study combined non-RRT- and RRT-directed therapies. In an unblinded pilot RCT involving 20 patients, Berthelsen et al. compared a forced fluid removal protocol in critically ill patients with fluid accumulation.^33^ The intervention arm of frusemide and as-required RRT resulted in an FB of −8434 mL compared to −641 mL with standard care. Of note, this study had a very low inclusion rate of 2%.

### 5.5 Fluid management protocols

Two studies investigated the feasibility and impact of a fluid management protocol on critically ill patients with AKI, as shown in Table 3.^34^^,^^35^ In non-RRT population, the REVERSE-AKI was a pilot RCT that compared usual care to RFP in 100 euvolemic, critically ill adults with AKI.^35^ The RFP resulted in an FB of −1148 mL at 72 h. Furthermore, an adjusted analysis suggested an association with less RRT in the restrictive fluid arm. In RRT population, the early dry cohort study was a before-and-after cohort study in 87 patients which investigated an RFP combined with perfusion-based adjustment of ultrafiltration in critically patients receiving continuous renal replacement therapy with >5% fluid overload.^34^ The intervention produced a 4292 mL difference in cumulative FB at day 5. Of note, despite the restrictive fluid input component of the intervention, the fluid input did not differ significantly between the groups.

**Table 3:** Fluid management protocols included studies.

Reference	Study Design	Country	Sample Size	AKI Definition	Population	RRT	Intervention	Comparator	Assessment of Fluid Status	Duration of Intervention	Difference in FB	Outcome Measures
Ruste 2022	SC, before-after cohort	France	87 (42/45)	Not provided. On CRRT.	ICU; CRRT; Fluid overload >5%	RRT as inclusion criteria	Restrictive fluid, perfusion-based protocol with Ufnet 2 mL/kg/hr.	Standard care	Body weight or fluid balance	5 days or ‘dry weight'	Cumulative fluid balance day 5 or discharge (- 7784 mL vs - 3492; p = 0.04)	CFB (as shown); Weight-adjusted FB (−7% vs −4.6%; p = 0.03); Mortality at day 30 (38% vs 45%; p = 0.58); RRT-free days at day 30 (10 days vs 11 days; p = 0.73)
Vaara 2021	MC Pilot RCT	Europe & Australia	100 (50/50)	KDIGO	AKI, ICU <72hrs; No RRT	Non-RRT patients	Restrictive fluid management	Standard care	Fluid balance	72 h	FB at 72 h (−1080 mL vs 61 mL; p = 0.033)	CFB (as shown); Duration of AKI (2 days vs 3 days; p = 0.071); RRT (13% vs 30%; p = 0.04)

Abbreviations: AKI = acute kidney injury; SC = single centre; MC = multicentre; RCT = randomised controlled trial; CRRT = continuous renal replacement therapy; RRT = renal replacement therapy; ICU = intensive care unit; FB = fluid balance.

## 6 Discussion

### 6.1 Key findings

The extent of the literature related to the scoping review question was limited, with only 22 studies included. Of these, 16 focused on RRT patients, only five studies examined non-RRT patients, and just one study included both. Of note, only nine of the studies were prospective interventional trials and 12 studies were conducted at multiple centres, highlighting the finite amount of existing research into the fluid management of critically ill patients with AKI. Furthermore, there was limited application of a comprehensive fluid management protocol that manipulated multiple components of FB, with only two included studies. Overall, the current literature did not demonstrate a consensus approach or a defining clinical trial to guide clinical practice. Moreover, this scoping review to assess the evidence for the management of FB demonstrated several knowledge gaps in the current literature.

### 6.2 Fluid input manipulation

There are insufficient studies and data to form conclusions on the manipulation of fluid input in the study population. Though four studies, two specific and two comprehensives, examined fluid input, the best approach to optimise fluid input remains uncertain. The two trials which focused on fluid input were limited in their applicability with their narrow focus on stroke volume variation and albumin administration. In the fluid management protocol trials, the fluid restriction component had an uncertain impact. In Ruste's study,^34^ there was no difference in fluid input with the RFP. In Vaara's study, there were no quantitative data available; however, the graphical representation of fluid input demonstrates a reduction in fluid input.^35^ Future research should first determine which fluid input variables, such as source, quantity, and type, are associated with positive FB. These findings should then be used to guide the development of future interventions surrounding RFP.

### 6.3 Enhancing fluid removal

Most of the literature focused on fluid removal and was informative in guiding future research. First, in non-RRT patients, the focus was on frusemide, which has an established role in the management of fluid overload, and the demonstrated trials suggest diuretics would be a key component of future investigations related to FB adjustment. The role of other diuretics or fenoldopam remains uncertain and should also be explored.

Fluid removal in the RRT population was a major focus of this review. The studies which indirectly examined FB are informative in that RRT intensity, downtime, and time to initiation need to be considered when addressing FB management. Naturally, NUF rates are integrally related to FB, and the demonstrated association with adverse patient-centred outcomes warrants further investigation on its own. The relationship between NUF, FB management, and outcomes is complicated. Observational evidence suggests a benefit with more negative daily FB, which attenuates but does not negate the association between high NUF rates and more adverse outcomes. Future research is required to determine how to balance competing priorities of potential harm from high NUF rates and the possible benefit of managing fluid overload.

Lastly, the Berthelsen study is noteworthy as the forced fluid removal intervention used furosemide and then ultrafiltration titration, making it the only study to address fluid management across the spectrum of severity of AKI.^33^ Previous research has demonstrated that the degree of fluid accumulation prior to RRT commencement is associated with negative outcomes; therefore, minimising fluid accumulation in AKI patients, as attempted by Berthelsen et al., warrants further investigation. Though the low-recruitment rate limits the interpretation of outcomes, future research should consider a similar approach to critically ill patients with AKI as opposed to automatically separating patients based on RRT status.

### 6.4 Fluid management protocol

The application of an RFP, which manipulates both input and output, has not been widely studied. The two studies included, those by Vaara^35^ and Ruste,^34^ have both demonstrated the feasibility and safety of this approach in the RRT and non-RRT AKI population, respectively. A comprehensive approach to fluid management including all contributors to FB has face value. Future research should first focus on the impact and potential benefit of each component of a comprehensive plan. A better understanding will allow resources to be directed towards measures, which are more likely to influence cumulative FB. Furthermore, the study by Vaara was unique in the included articles as the approach was to avoid fluid accumulation after resuscitation of the participants as opposed to treating fluid overload.^35^ Given the potential harms of fluid overload, this approach is noteworthy, and future research should explore the relationship between fluid overload prevention and outcomes.

### 6.5 Strengths and limitations of the scoping review

This study had several strengths. First, we systematically conducted this scoping review utilising standardised Joanna Briggs Institute methodology which led to a thorough, unbiased review of the current literature. Second, our review contains contemporary literature with articles from 2012 onwards included which enhances the applicability to current practice and acts as a foundation to guide future research. Though there has been no clear transition in the clinical practice of fluid management in the past, 2012 was used as this represents the last 10 years of critical care research and coincides with the publication of the standardised KDIGO definition of AKI. Third, our review contains a wide breadth of potential interventions related to the fluid management of critically ill patients with AKI, which is a representation of the complex, multifaceted nature of the problem. This approach has facilitated a holistic description of current fluid management practice and research in critically ill patients with AKI.

Though a systematic approach was taken to conduct this scoping review, there are some limitations to be acknowledged. The scoping review examined the literature related to critically ill patients with AKI as opposed to all patients with a critical illness. It is possible that literature related to the FB management of all critically ill patients may have provided insights into prioritising future research priorities in the AKI population. The scope of the review may have prevented the inclusion of such concepts and ideas, which would be applicable to the population of interest. However, patients with AKI are a specific and unique population in relation to fluid management and data from a general population of critically ill patients may not apply to them.

The scoping review did not produce a synthesised result to a particular question which would guide future clinical practice. This review is limited in its ability to impact the care of critically ill patients with AKI. However, we believe the scope of the evidence uncovered in the conduct of this review demonstrates the lack of feasibility to conduct such a direct systematic review. Furthermore, given the aims of the review, an assessment of methodological limitations or risk of bias of the evidence included was not performed. Though in keeping with scoping review standard, this needs to be taken into consideration when considering the individual studies included.

## 7 Conclusion

Despite the volume of observational evidence demonstrating potential harm with fluid accumulation in critically ill patients with AKI, there is limited evidence examining the impact of different management strategies related to FB. The scoping review demonstrated insufficient evidence to guide clinical practice; however, it has highlighted several knowledge gaps where future research should be prioritised.

## Funding

No funding was provided in the conduct of this scoping review.

## Conflicts of interest

There is no conflict of interest in this project.

## Appendix I Search strategy

MEDLINE via EBSCOhost

#1

AB (“fluid balance” OR “fluid input” OR “urine” OR “oliguria” OR (MH “Oliguria”) OR “fluid overload” OR “fluid management” OR (MH “Water-Electrolyte Imbalance+”) OR (MH “Water-Electrolyte Balance+”) OR “goal-directed fluid therapy” OR “diuretics” OR (MH “Diuretics+”) OR “furosemide” OR “frusemide” OR “restrictive fluid management”) OR TI (“fluid balance” OR “fluid input” OR “urine” OR “oliguria” OR (MH “Oliguria”) OR “fluid overload” OR “fluid management” OR (MH “Water-Electrolyte Imbalance+") OR (MH “Water-Electrolyte Balance+”) OR “goal-directed fluid therapy” OR “diuretics” OR (MH “Diuretics+” OR “furosemide” OR “frusemide” OR “restrictive fluid management”)

#2

AB ((MH “Acute Kidney Injury+”) OR (MH “Continuous Renal Replacement Therapy”) OR (MH “Renal Replacement Therapy+”) OR (MH “Intermittent Renal Replacement Therapy”) OR (MH “Hybrid Renal Replacement Therapy”) OR (MH “Creatinine”) OR “acute kidney injury” OR “acute renal failure” OR “acute renal injury” OR “acute kidney failure” OR “kidney injury” OR “creatin∗” OR “dialysis” OR “continuous renal replacement therapy” OR “renal replacement therapy” OR “intermittent hemodialysis”) OR TI ((MH “Acute Kidney Injury+”) OR (MH “Continuous Renal Replacement Therapy”) OR (MH “Renal Replacement Therapy+”) OR (MH “Intermittent Renal Replacement Therapy”) OR (MH “Hybrid Renal Replacement Therapy”) OR (MH “Creatinine”) OR “acute kidney injury” OR “acute renal failure” OR “acute renal injury” OR “acute kidney failure” OR “kidney injury” OR “creatin∗” OR “dialysis” OR “continuous renal replacement therapy” OR “renal replacement therapy” OR “intermittent hemodialysis”)

#3

AB ((MH “Critical Care+”) OR (MH “Critical Illness”) OR (MH “Shock+”) OR “critical care” OR “critical illness” OR “shock” OR “intensive care” OR “ICU” OR “intensive care unit” OR “intensive” OR “critically ill” OR “critical illness” OR “shock”) OR TI ((MH “Critical Care+”) OR (MH “Critical Illness”) OR (MH “Shock+”) OR “critical care” OR “critical illness” OR “shock” OR “intensive care” OR “ICU” OR “intensive care unit” OR “intensive” OR “critically ill” OR “critical illness” OR “shock”)

Search - #1 AND #2 AND #3

Limiters – Date of Publication 20120101-

Expanders - Apply equivalent subjects

Search modes - Boolean/Phrase

EMBASE

#1

(‘fluid balance’/exp OR ‘fluid balance’ OR ‘fluid input’ OR ‘urine'/exp OR ‘urine’ OR ‘oliguria’/exp OR ‘oliguria’ OR ‘fluid overload’/exp OR ‘fluid overload’ OR ‘fluid management’ OR ‘goal-directed fluid therapy’ OR ‘diuretics’/exp OR ‘diuretics’ OR ‘furosemide’/exp OR ‘furosemide’ OR ‘frusemide’/exp OR ‘frusemide’ OR ‘restrictive fluid management':ab,ti) AND [2012–2022]/py AND [embase]/lim NOT ([embase]/lim AND [medline]/lim)

#2

(‘acute kidney injury’/exp OR ‘acute kidney injury’ OR ‘acute renal failure’/exp OR ‘acute renal failure’ OR ‘acute renal injury’/exp OR ‘acute renal injury’ OR ‘acute kidney failure’/exp OR ‘acute kidney failure’ OR ‘kidney injury’/exp OR ‘kidney injury’ OR ‘creatin∗’ OR ‘dialysis’/exp OR ‘dialysis’ OR ‘continuous renal replacement therapy’/exp OR ‘continuous renal replacement therapy’ OR ‘renal replacement therapy’/exp OR ‘renal replacement therapy’ OR ‘intermittent hemodialysis’/exp OR ‘intermittent hemodialysis':ab,ti) AND [2012–2022]/py AND [embase]/lim NOT ([embase]/lim AND [medline]/lim)

#3

(‘critical care’/exp OR ‘critical care’ OR ‘intensive care’/exp OR ‘intensive care’ OR ‘icu’ OR ‘intensive care unit’/exp OR ‘intensive care unit’ OR ‘intensive’ OR ‘critically ill’/exp OR ‘critically ill’ OR ‘critical care medicine’/exp OR ‘critical illness’/exp OR ‘critical illness’ OR ‘shock’/exp OR ‘shock’:ab,ti) AND [2012–2022]/py AND [embase]/lim NOT ([embase]/lim AND [medline]/lim)

Search - #1 AND #2 AND #3

CINAHL via EBSCOhost

#1

AB (“fluid balance” OR “fluid input” OR “urine” OR “oliguria” OR (MH “Oliguria”) OR “fluid overload” OR “fluid management” OR (MH “Water-Electrolyte Imbalance+”) OR (MH “Water-Electrolyte Balance+”) OR “goal-directed fluid therapy” OR “diuretics” OR (MH “Diuretics+”) OR “furosemide” OR “frusemide” OR “restrictive fluid management”) OR TI (“fluid balance” OR “fluid input” OR “urine” OR “oliguria” OR (MH “Oliguria”) OR “fluid overload” OR “fluid management” OR (MH “Water-Electrolyte Imbalance+”) OR (MH “Water-Electrolyte Balance+”) OR “goal-directed fluid therapy” OR “diuretics” OR (MH “Diuretics+”) OR “furosemide” OR “frusemide” OR “restrictive fluid management”)

#2

AB ((MH “Acute Kidney Injury+”) OR (MH “Continuous Renal Replacement Therapy”) OR (MH “Renal Replacement Therapy+”) OR (MH “Intermittent Renal Replacement Therapy”) OR (MH “Hybrid Renal Replacement Therapy”) OR (MH “Creatinine”) OR “acute kidney injury” OR “acute renal failure” OR “acute renal injury” OR “acute kidney failure” OR “kidney injury” OR “creatin∗” OR “dialysis” OR “continuous renal replacement therapy” OR “renal replacement therapy” OR “intermittent hemodialysis”) OR TI ((MH “Acute Kidney Injury+”) OR (MH “Continuous Renal Replacement Therapy”) OR (MH “Renal Replacement Therapy+”) OR (MH “Intermittent Renal Replacement Therapy”) OR (MH “Hybrid Renal Replacement Therapy”) OR (MH “Creatinine”) OR “acute kidney injury” OR “acute renal failure” OR “acute renal injury” OR “acute kidney failure” OR “kidney injury” OR “creatin∗” OR “dialysis” OR “continuous renal replacement therapy” OR “renal replacement therapy” OR “intermittent hemodialysis”)

#3

AB ((MH “Critical Care+”) OR (MH “Critical Illness”) OR (MH “Shock+”) OR “critical care” OR “critical illness” OR “shock” OR “intensive care” OR “ICU” OR “intensive care unit” OR “intensive” OR “critically ill” OR “critical illness” OR “shock”) OR TI ((MH “Critical Care+”) OR (MH “Critical Illness”) OR (MH “Shock+”) OR “critical care” OR “critical illness” OR “shock” OR “intensive care” OR “ICU” OR “intensive care unit” OR “intensive” OR “critically ill” OR “critical illness” OR “shock”)

Search - #1 AND #2 AND #3

Limiters – Date of Publication 20120101-; Exclude MEDLINE records

Expanders - Apply equivalent subjects

Search modes - Boolean/Phrase
